# Early detection of idiopathic thoracic ventral spinal cord herniation by using imaging: A case report

**DOI:** 10.1002/ccr3.8112

**Published:** 2023-11-09

**Authors:** Mana Kaida, Hirohito Hirata, Hikaru Noda, Yuya Kishikawa, Tomohito Yoshihara, Takaomi Kobayashi, Masatsugu Tsukamoto, Masaaki Mawatari, Tadatsugu Morimoto

**Affiliations:** ^1^ Faculty of Medicine Saga University Saga Japan; ^2^ Department of Orthopedic Surgery, Faculty of Medicine Saga University Saga Japan

**Keywords:** gait disturbance, overlooked case, spinal cord herniation, thoracic vertebrae

## Abstract

In middle‐aged and older populations, clinicians often suspect lumbar spine disease when the gait is disturbed with lumbar lower extremity numbness, but spinal herniation at the thoracic level may be causal. Early detection, appropriate treatment, and minimization of complications requires understanding of characteristic magnetic resonance imaging findings of herniation.

## Graphical abstract

Sagittal T2‐weighted MRI showing anterior deviation and flexion of the spinal cord: Spinal cord herniation.

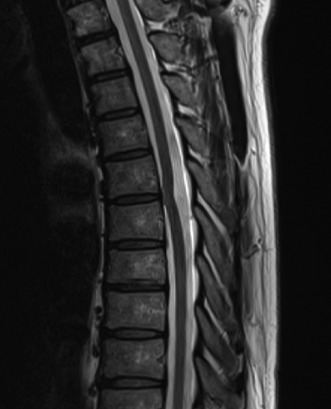

## 1 INTRODUCTION

Hernias are well‐recognized medical conditions involving the protrusion of organs or tissues through weakened areas of the surrounding structures. Among the various types of hernias, inguinal, cerebral, and intervertebral disc herniations have been extensively studied and documented in the medical literature. However, idiopathic spinal cord herniation (ISCH) remains a rare and intriguing condition. ISCH was first described by Wortzman et al.^1^ in 1974 as a ventral dural defect extending to the adjacent epidural space, usually at the thoracic level.^2^

ISCH is usually more common in older and middle‐aged patients with lower extremity symptoms and is often misdiagnosed and mistreated as a lumbar spine disorder.^3^, ^4^, ^5^

Here, we present a surgical video and characteristic pre‐ and postoperative images of a patient with T6/7‐level ISCH, who was treated with surgical repair of the dural defects. This case report aims to address the diagnostic issues of this disease. It also emphasizes the need for early diagnosis, using characteristic imaging features, and treatment, which are critical to improve outcomes. In addition, we aimed to strengthen the knowledge base of ISCH by surgically reviewing the lesions in this unique case.

## 2 CASE REPORT

### 2.1 Case history and examination

**FIGURE 1:**
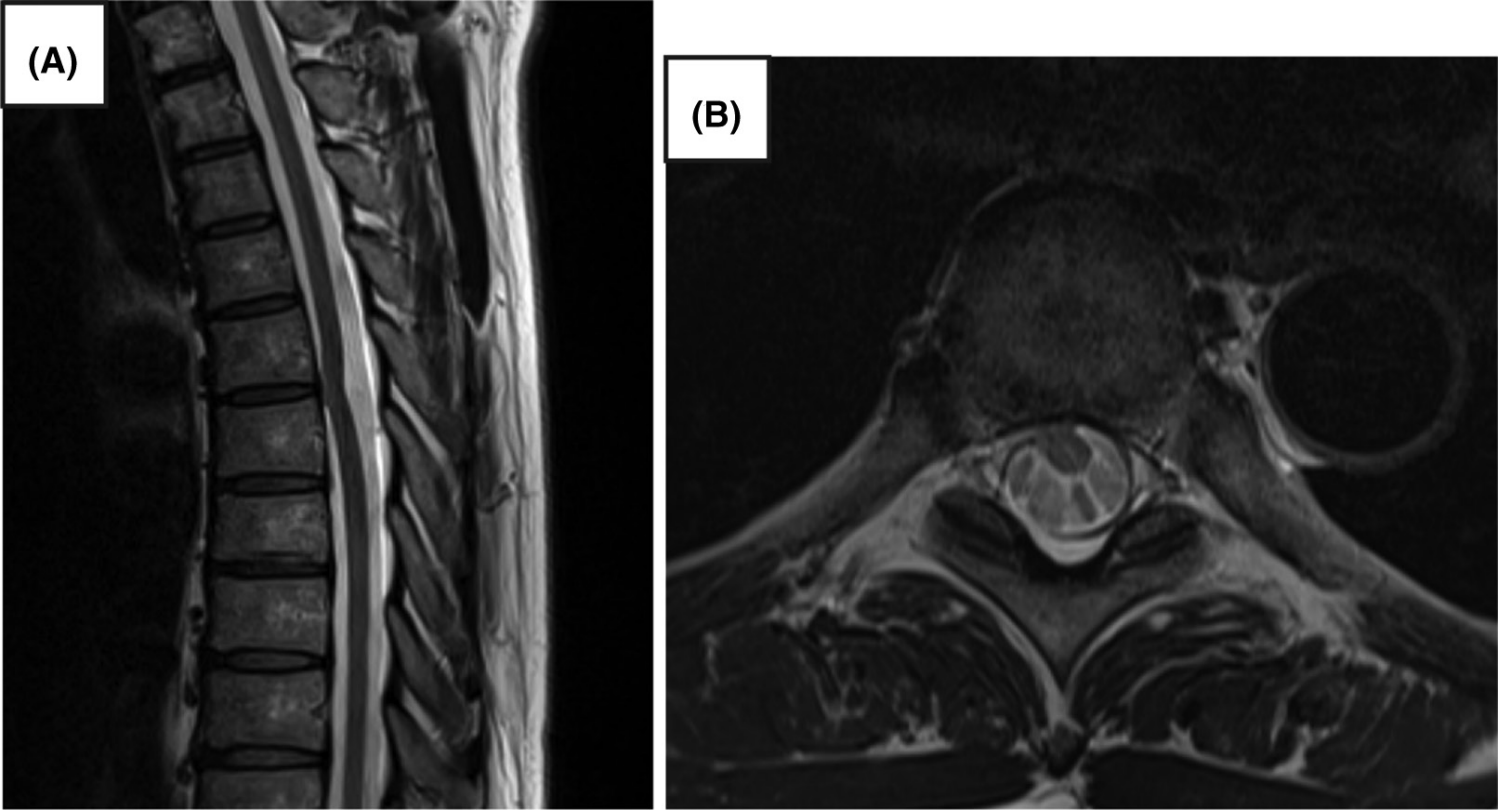
Preoperative magnetic resonance imaging (MRI). (A) Sagittal T2‐weighted MRI showing anterior deviation and flexion of the spinal cord at the Th6/7 level and loss of the spinal fluid space anterior to the spinal cord. (B) Axial T2‐weighted MRI showing ventral deviation of the spinal cord at the Th6/7 level.

**FIGURE 2:**
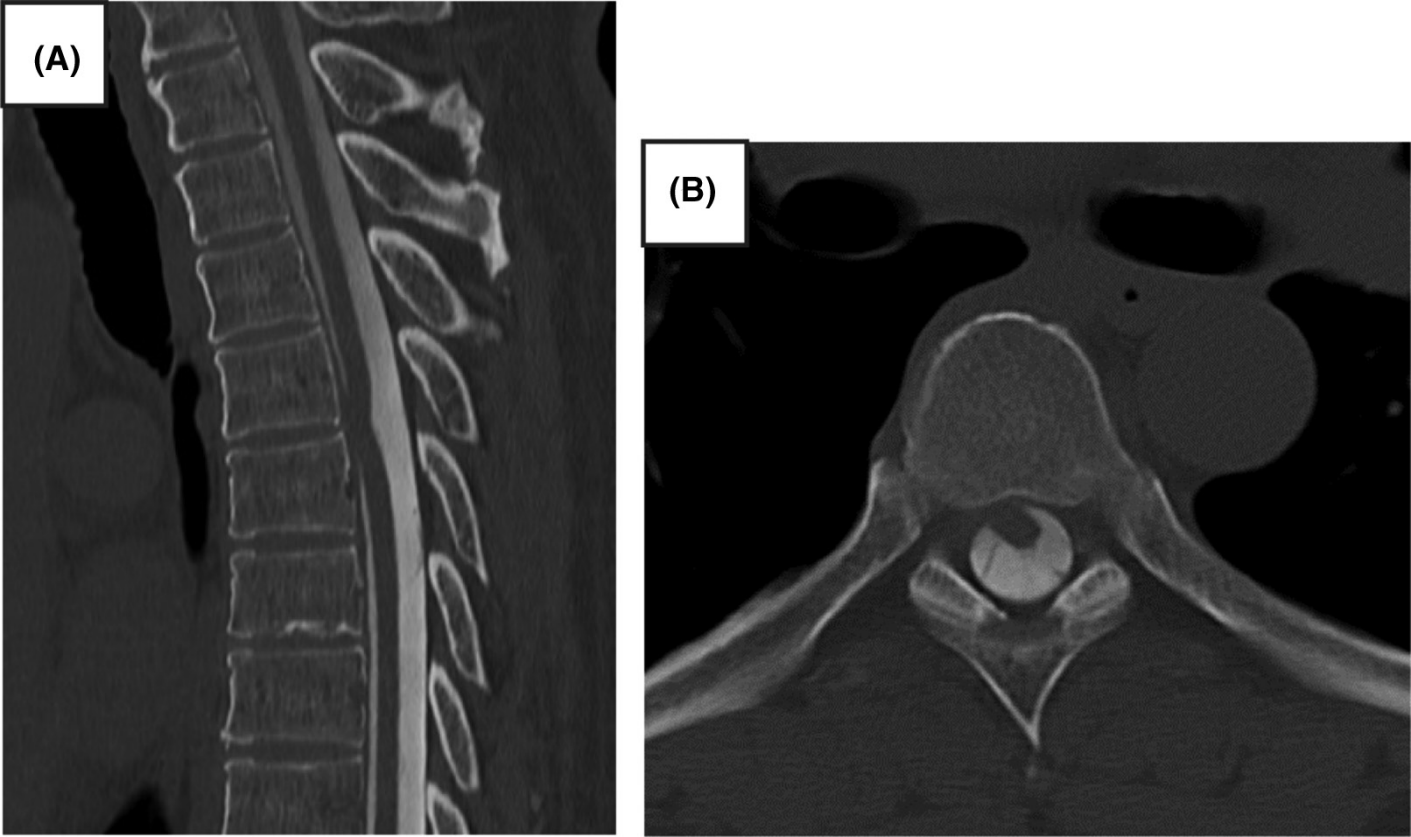
Preoperative computed tomography (CT) myelogram. (A) Sagittal CT myelography showing anterior deviation and flexion of the spinal cord at the Th6/7 level. (B) Axial CT myelogram showing ventral deviation of the spinal cord at the Th6/7 level.

A 55‐year‐old Japanese man presented to our hospital with persistent symptoms of low back pain and right lower extremity numbness of 1‐year duration, which had been diagnosed elsewhere as lumbar spinal canal stenosis. He had no medical history, spinal surgery, medications, or family history. Although pain was absent, numbness was present throughout both lower extremities, and he had right lower extremity weakness, difficulty standing on one leg, spastic gait disturbance, and hyperreflexia of the lower extremity deep reflexes. Thus, we suspected thoracic spinal cord disorder rather than lumbar spine disease. Sagittal magnetic resonance imaging (MRI) showed focal ventral and rightward displacement, sharp angulation of the spinal cord, and enlargement of the dorsal subarachnoid space of the spinal cord at the T6/7 level (Figure 1A,B). Computed tomography (CT) myelography showed ventral deviation of the spinal cord (Figure 2A,B). The patient was diagnosed with ISCH at T6/7.

After an explanation, the patient provided informed consent for surgical treatment of ISCH without medical therapy.

### 2.2 Treatment

**FIGURE 3:**
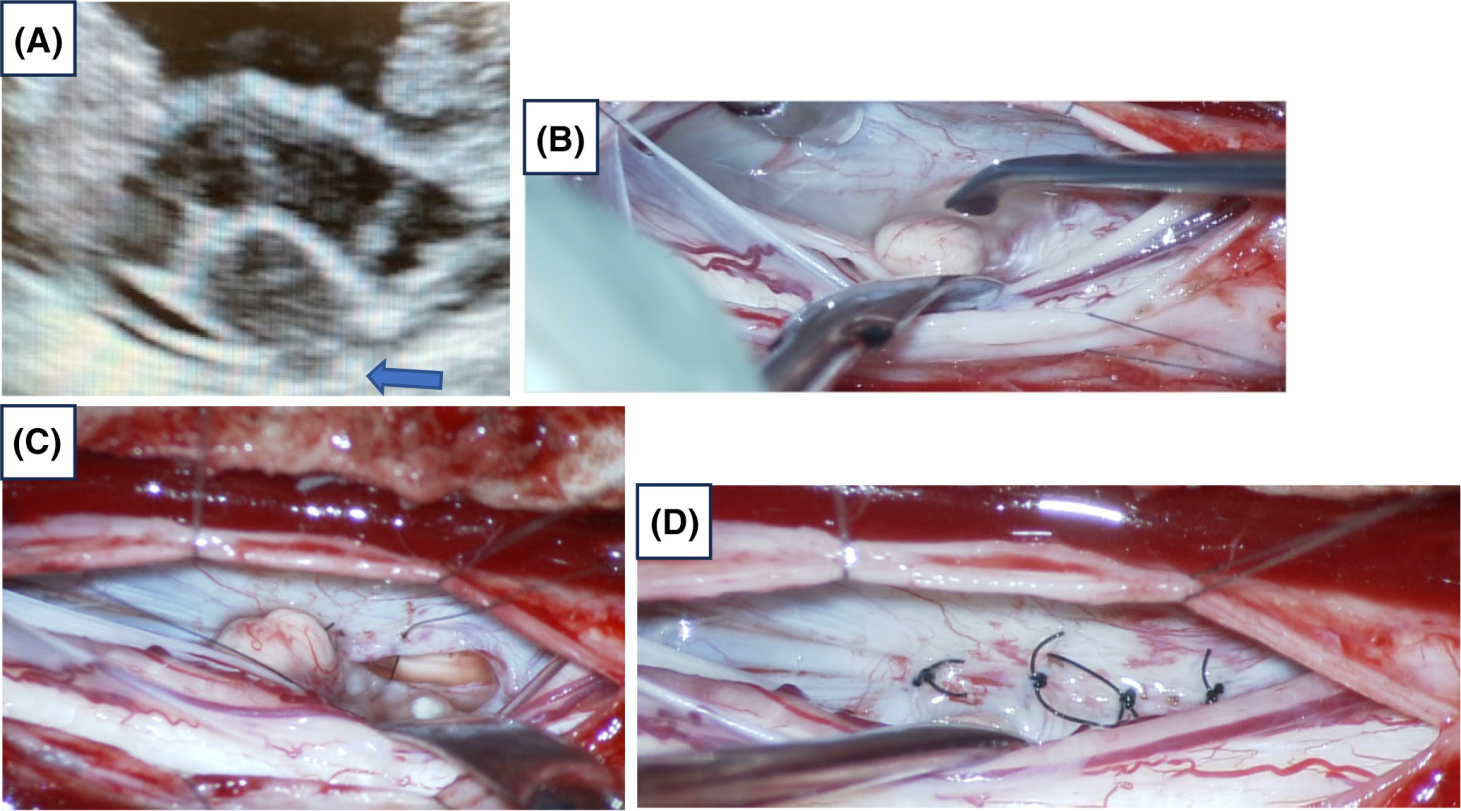
(A) Intraoperative ultrasound confirming that the spinal cord deviates ventrally and the protruding hernia looks like a “navel” (arrow). (B) Surgical photograph: A part of the spinal cord (navel‐like structure) fitting into the dural defect is being repositioned. (C) Surgical photograph: The dural defect is repaired with double dura mater, with suturing on only the inner layer. (D) Surgical photograph: The dural defect is suture‐repaired to prevent the spinal cord from protruding back through the dural defect.

After a T6 and T7 laminectomy was performed, ultrasonography showed that the spinal cord deviated to the right ventral side and that part of the spinal cord protruded ventrally, akin to a “navel” (Figure 3A).

The spinal cord was attached to the ventral dura mater (Figure 3B). The spinal cord was retracted centrally after releasing the adhesions at the spinal dural defect. An oval defect in the ventral dura mater, from which the ventral portion of the spinal cord protruded (Figure 3C), was noted. The outer layer of the dura was intact throughout the defect, confirming that rupture of the inner dural membrane had occurred. The defect was sutured and repaired so that the spinal cord would not attach to or protrude from the dural defect (Figure 3D).

### 2.3 Outcome and follow‐up

**FIGURE 4:**
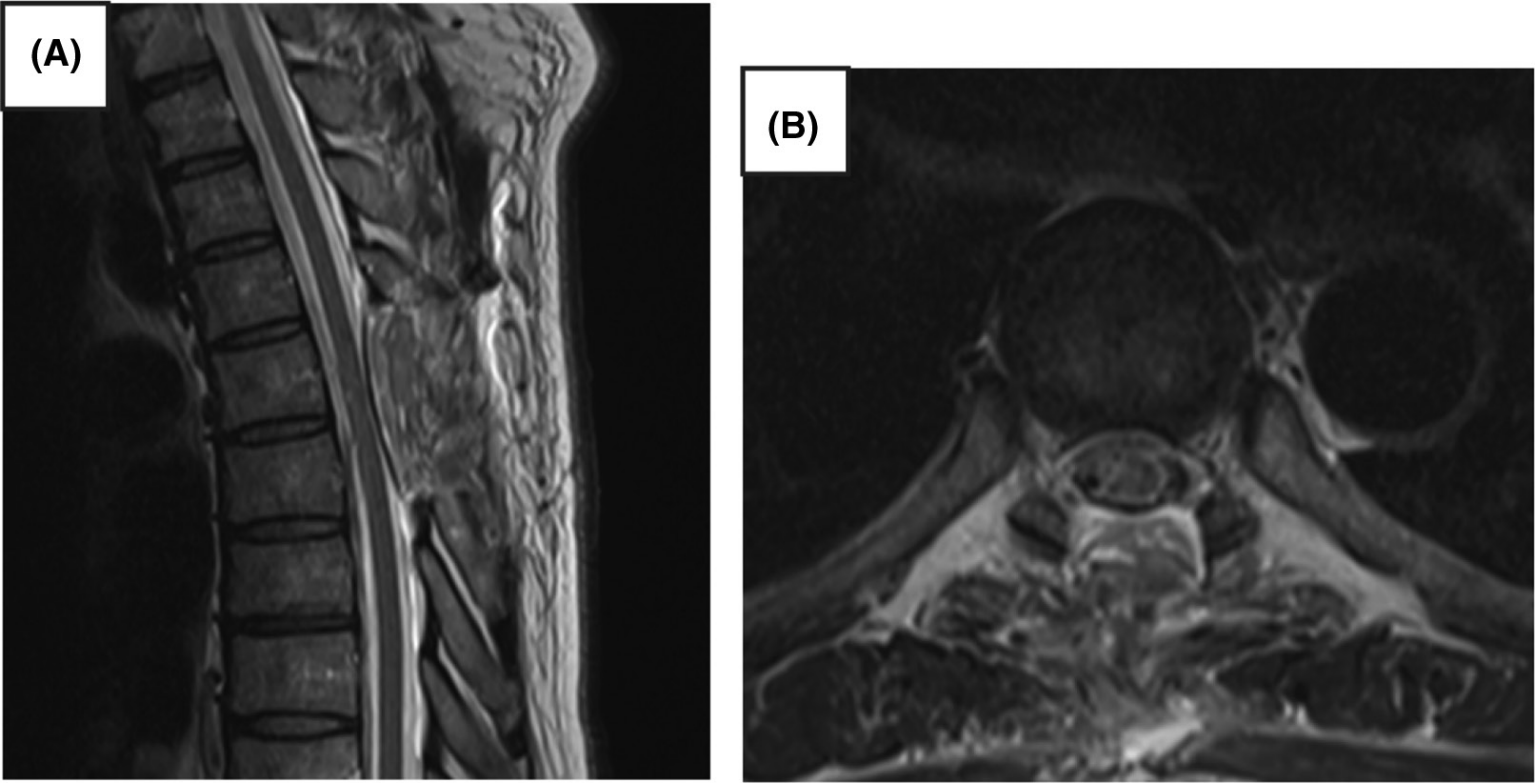
T2‐weighted magnetic resonance image obtained at 1‐week postoperatively, showing the normal position of the spinal cord: (A) Sagittal, (B) Axial.

MRI at 1‐week postoperatively showed that the spinal cord was repositioned to its normal position (Figure 4A,B). The patient experienced no change in sensory deficits, but muscle strength had gradually recovered by months postoperatively.

## 3 DISCUSSION

### 3.1 Neurological symptoms of ISCH

Some ISCH patients have neurological symptoms. Yang et al.^3^ reported on 96 men and 156 women with a mean age of 51.6 ± 12.1 years (range: 20–78 years), with ISCH at a thoracic level (central [T5–T8]: 66.0%; upper [T1–T4]: 30.0%; and lower [T9–T12]: 4.0%), and with a mean preoperative symptom duration of 4.2 ± 4.5 years (range: 0.1–32 years). The symptoms of ISCH included Brown–Séquard syndrome (58.6%, *n* = 139/237), myelopathy (31.2%, *n* = 74/237), spastic paraparesis (13.9%, *n* = 33/237), and radiculopathy (1.3%, *n* = 3/237). Brown–Séquard syndrome is a neurological syndrome that results from hemisection of the spinal cord. Weakness, paralysis, and proprioceptive deficits occur on the ipsilateral side of the lesion, and pain and temperature sensation are lost on the contralateral side.^6^

Although the etiology of ISCH remains unknown, several reports have proposed that the spinal cord is injured when it is shifted relatively ventrally by thoracic kyphosis. It becomes trapped in ventral and lateral defects in the dura mater caused by congenital or microtrauma, due to factors such as respiration, body movement, or spinal fluid pressure.^3^ Because the spinal cord often becomes trapped ventrally or laterally, symptomatic presentation is often that of Brown–Séquard syndrome.^3^

As ISCH is more common in middle‐aged and older patients and mainly presents with lower‐extremity symptoms, lumbar degenerative spinal disease is typically suspected, as in the present case. As lumbar spine degeneration is frequently observed on lumbar spine MRI in this population, ISCH is underdiagnosed and is often misdiagnosed as lumbar degenerative spinal cord disease.^3^, ^4^ Differential diagnoses of ISCH are dorsal arachnoid cyst, astrocytoma, disc herniation, extradural compression, and transverse myelitis.^3^

### 3.2 Characteristic imaging findings of ISCH

MRI is a valuable noninvasive tool for diagnosis of ISCH. ISCH presents characteristic imaging findings, such as focal ventral displacement and sharp angulation of the spinal cord, sometimes accompanied by anteroposterior thinning of the spinal cord in this region.^4^, ^7^ In our case, enlargement of the dorsal subarachnoid space of the spinal cord was also noted.

Myelography performed under fluoroscopic guidance with immediate CT myelography conducted once an intrathecal contrast medium is administered, is used to confirm the diagnosis in uncertain cases. In CT myelography, the spinal cord cavity is contrasted, making the lesion easier to identify.

Subtle signal changes in the ventral epidural space on high‐resolution T2 MRI can sometimes indicate a herniation, which is difficult to detect. In our case, the intraoperative findings of a “navel”‐shaped, degenerative, spherical, bulging ventral hernia of the spinal cord was not discernible on preoperative MRI or CT, but was discernible on intraoperative echocardiography. Thus, intraoperative ultrasonography is also useful for identifying the location of the hernia.^3^

### 3.3 Management of ISCH

According to Aizawa et al.,^7^ ISCH can be classified into the following three types, according to the form of spinal cord prolapse: The first is the direct prolapse type, in which the spinal cord prolapses directly into the epidural space; the second is the double dural type, in which the spinal cord prolapses between the inner and outer layers of the double dura mater; and the third is the epidural cyst type, in which an epidural cyst is present into which the spinal cord prolapses. On imaging examination, distinguishing between the direct prolapse and double dural types may be difficult. In both types, the atrophied and deformed spinal cord is depicted as touching the ventral or ventral–lateral side of the dural canal, and is seen as continuous with the epidural space. If a preoperative diagnosis is difficult, the morphology of the spinal cord prolapse can be confirmed intraoperatively, to determine the operative technique. In the treatment of the direct prolapse type, adhesions are detached and repositioned, and then dural sutures or duraplasty are performed to prevent spinal fluid leakage. Double dural epidural cysts do not require dural suturing or duraplasty. After detachment and repair of the adhesions, hernia foramen magnification is performed to enlarge the hernia foramen cephaloposteriorly to prevent re‐entrapment in the hernia.

In Yang et al.'s review of 209 cases, 136 were treated with graft or patch duraplasty, 56 with defect enlargement, and 17 with primary suture closure.^3^ In some cases, hernias recurred due to defect enlargement.^8^ In this case, a temporary repair was performed because the hernia was of the direct‐prolapse type and could be sutured relatively easily. The patient was able to walk with reduced numbness in the lower extremities soon after surgery.

ISCH is a treatable spinal cord disease. The shorter the disease duration, the better is the outcome. Conversely, the longer the disease duration, the worse is the outcome.^9^, ^10^ Therefore, early diagnosis and appropriate treatment are crucial.

### 3.4 Reminder to clinicians

ISCH is characterized by the presence of Brown–Séquard syndrome symptoms.^9^, ^10^ ISCH is generally thought to be characterized by an early onset of paresthesia, followed by slow progression of myelopathy. However, symptoms other than myelopathy may be observed early in the course of the disease, because of the variable degrees of spinal herniation at the site of the dural defect. Therefore, in clinical practice, ISCH should be considered as a disease with a variety of manifestations.^3^

ISCH is a spinal cord disorder affecting the thoracic spine, with poor blood flow. However, it is a treatable condition, and early diagnosis and treatment can affect prognosis. Thus, clinicians should aggressively and promptly evaluate suspected ISCH. This is accomplished by recalling lesions at the thoracic level through a thorough clinical evaluation and recognizing the characteristic imaging findings, to facilitate accurate diagnosis, and determining the appropriate course of treatment.

## 4 CONCLUSION

ISCH is a rare disease that predominantly affects the middle thoracic spinal cord and involves herniation of the spinal cord through a dural defect. While it may present with symptoms of Brown–Séquard syndrome, it may not present with typical symptoms because of its gradual progression. As in the present case, the condition is often misdiagnosed as a lumbar spine disorder, such as sciatica. Imaging is important for accurate diagnosis, and high‐resolution T2 images are particularly useful for the diagnosis of ISCH. MRI sagittal sections show focal ventral displacement, sharp angulation of the spinal cord, and enlargement of the dorsal subarachnoid space of the spinal cord. Treatment is surgical. In the present case, the patient underwent dural suturing because of a direct prolapse. As ISCH is treatable, early detection using diagnostic imaging is important.

## AUTHOR CONTRIBUTIONS

**Mana Kaida:** Data curation; writing – original draft. **Hirohito Hirata:** Investigation; methodology; validation; visualization. **Hikaru Noda:** Data curation; resources; visualization. **Yuya Kishikawa:** Data curation; visualization. **Tomohito Yoshihara:** Data curation; visualization. **Takaomi Kobayashi:** Conceptualization; supervision; writing – review and editing. **Masatsugu Tsukamoto:** Data curation; validation. **Masaaki Mawatari:** Project administration; supervision; writing – review and editing. **Tadatsugu Morimoto:** Conceptualization; formal analysis; investigation; supervision; writing – review and editing.

## FUNDING INFORMATION

None.

## CONFLICT OF INTEREST STATEMENT

The authors declare that they have no competing interests.

## ETHICS STATEMENT

Not mandated for case reports.

## CONSENT

Written informed consent was obtained from the patient to publish this report in accordance with the journal's patient consent policy.

## Data Availability

Data pertaining to this case can be obtained by contacting the corresponding author.
